# The Impact of Customer Mistreatment on Employee Displaced Aggression: The Moderating Effect of Interpersonal Sensitivity and Moral Identity

**DOI:** 10.3389/fpsyg.2020.550978

**Published:** 2020-09-29

**Authors:** Fang Liu, Gang Chen, Yu Liu

**Affiliations:** ^1^ School of Business Administration, Southwestern University of Finance and Economics, Chengdu, China; ^2^ School of Public Affairs and Administration, University of Electronic Science and Technology of China, Chengdu, China

**Keywords:** customer mistreatment, interpersonal injustice, displaced aggression, interpersonal sensitivity, moral identity

## Abstract

Although customer mistreatment produces harmful consequences for employees and organizations, our understanding of the boundary conditions of customer mistreatment has largely been neglected. This study examines whether and when customer mistreatment influences employee displaced aggression toward coworkers by demonstrating interpersonal sensitivity and moral identity traits as two critical boundary conditions. Through the analysis of 623 employees’ questionnaire data, the results showed that customer mistreatment was positively related to employee displaced aggression toward coworkers. Furthermore, interpersonal sensitivity exacerbates the effect of customer mistreatment on displaced aggressive behaviors, while moral identity buffering the effect.

## Introduction

Many service-oriented enterprises believe that “Customer is God.” With the hope to improve customer satisfaction and loyalty through the service concept of “customer first,” employees are required by enterprises to maintain humble attitudes and behaviors in the process of service interaction with customers (Yagil, 2008), which put buyers and sellers in an unequal position, and to some extent, encourage the emergence and development of customer mistreatment. Studies have shown that in the process of service consumption, it is not rare for customers to be overly self-centered, belittle the identity of employees, as well as verbally abuse and excessively demand employees (Grandey et al., 2004). Unfortunately, such kinds of customer mistreatment toward employees have been ignored in the process of enterprises pursuing the maximization of customer satisfaction. Until recent years, with the rapid development of the service industry, the frequency of customer mistreatment of service employees at work has been increased significantly (Reynolds and Harris, 2006), and the attention to customer mistreatment and its adverse consequences has also been gradually increasing in the academic and practical circles. Based on the conservation of resources (CORs) theory, as a stressor in employees’ working environment, customer mistreatment often consumes a large amount of psychological resources of employees, causing psychological pressure, negative emotions, and emotional exhaustion, impairing employee well-being (Sommovigo et al., 2019b), as well as affecting their physical and mental health (Almeida, 2005; Grandey et al., 2007). These negative effects may further lead to higher generalized customer-directed incivility intentions (Sommovigo et al., 2020), lower service recovery performance (Sommovigo et al., 2019a), and unethical behaviors of employees, such as service sabotage and displaced aggressive behaviors (Wang et al., 2011; Koopmann et al., 2015; Liu et al., 2015), which will have adverse effects on service relationship and enterprises operation.

However, the current existing research on the effects of customer mistreatment mostly focuses on employees’ service sabotage to customers, and their organizational withdrawal and job performance (Boswell and Olson-Buchanan, 2004; Wang et al., 2011; Baranik et al., 2017; Sommovigo et al., 2019b); there is little research on the influence of employees on their coworkers. Hence, this study attempts to explore the relationship between customer mistreatment and employees’ aggressive behaviors toward coworkers, that is, displaced aggression toward coworkers. One of the mechanisms for the formation of aggressive behaviors is the self-regulation disorder caused by resource consumption. Employees who suffer from customer mistreatment consume a substantive amount of psychological resources. Such resources in this study are usually referred to as self-regulatory resources, which involve individuals’ mental efforts to control and alter naturally occurring emotions, behaviors, and mental states (Muraven and Baumeister, 2000; Baumeister and Vohs, 2003; Beal et al., 2005; Liu et al., 2015). As such, employees’ ability of self-regulation is impaired, and it is difficult for them to regulate and restrain their aggressive impulses, as consequence aggressive behaviors are more likely to occur (Liu et al., 2015). In other words, under the supervision of the organizational service rules, employees would be in fear of taking the organizational punishment and dare not to directly carry out reactive aggression toward customers. Instead, they might change the target of attack and turn to the innocent people around them, such as their coworkers, as such displaced aggression oriented toward coworkers is formed.

As employees’ behaviors are not only affected by the work situations, but their individual characteristics also play a vital role as an important internal cause. Therefore, when considering employees’ aggressive behaviors in unfair service situations, we should take into account employees’ individual characteristics, which also provide insights for resolving the adverse consequences caused by customer mistreatment. Interpersonal sensitivity, as a kind of personality trait, refers to undue and excessive awareness of, and sensitivity to, the behaviors and feelings of others (Boyce and Parker, 1989). This trait makes an individual overly sensitive to interpersonal relationship, especially to criticism or rejection (Boyce and Parker, 1989). Employees with different interpersonal sensitivities usually have different perceptions of customer unkindness and harm. Based on the CORs theory, employees with high interpersonal sensitivity bear more pressure, consume more psychological resources, and are prone to the phenomenon of reduction or even failure in self-control performance. Therefore, interpersonal sensitivity, as an individual difference variable, may adjust the impact of customer mistreatment on employee displaced aggression. In addition, moral identity is also an individual characteristic variable, and it is defined as a self-conception organized around a set of moral traits (Aquino and Reed, 2002), such as honesty, kindness, and willingness to help others, reflecting the degree of importance of moral traits in individual self-evaluation (Zhang and Liao, 2017). According to the social cognitive theory (SCT), moral identity plays an important normative role in the formation of individual behaviors, which are conducive to the self-restraint of individuals and the suppression of immoral behaviors (Aquino et al., 2009). This indicates that moral identity may have a moderating effect on the relationship between customer mistreatment and employee displaced aggression.

In this study, participants were recruited from the frontline service employees in China to examine the relationship between customer mistreatment and employee displaced aggression toward coworkers, and to explore the intervention mechanism of customer mistreatment from the perspective of individual characteristics, in an attempt to put forward the defense and adjustment measures of customer mistreatment suitable for the situations in China. This study aims to make contributions in the following three aspects. First, based on the CORs theory, the relationship between customer mistreatment and employee displaced aggression toward colleagues is empirically examined, which helps to clarify the influence process of customer behavior on employee behavior. Second, exploring the moderating effect of interpersonal sensitivity and moral identity on the relationship between customer mistreatment and employee displaced aggression in light of COR theory and SCT, respectively. Finally, trying to provide a new perspective for the intervention research of customer mistreatment, as well as enrich and develop the practical application of individual characteristic difference variables in the practical management of customer mistreatment. The rest of this paper is organized as following: (1) In the second section, we propose the three hypotheses of this paper by reviewing the existing literature, as well as constructing the research model. (2) The third section describes the materials and methods, including participants and measures. (3) The fourth section presents the statistical analyses and reports the empirical results. (4) The final section shows research conclusions and discusses theoretical implications, practical implications, as well as limitations and future research direction.

## Theoretical Framework and Hypothesis Development

### Customer Mistreatment and Employee Displace Aggression

Customer mistreatment refers to the “low-quality” interpersonal treatment that the employees perceive from their customers during service interactions, such as verbal insults, unfair demands, and disrespect behaviors (Wang et al., 2013), which often cause employees to experience strong negative emotions or work stress (Grandey et al., 2004). However, according to the service requirements of the organization, employees should maintain a sincere, friendly, and polite service attitude when facing mistreatment from customers. It requires consuming more psychological resources for emotional suppression, thought suppression, and attention control in order to show the emotions and attitudes specified by the organization (Zapf, 2002), such as emotional labor.

In the process of emotional labor, large amounts of the individual’s psychological resources are consumed and cannot be supplemented from environment, further to activate protection mechanism (Hobfoll, 1989). It will consume more other resources of the individual to compensate for the consumption of the initial resources (i.e., secondary resource loss), while the secondary resources are easily lost and will cause the spiral of resource loss (Hobfoll, 2002), making the resources of the employee be lost or even exhausted in a short time. When people’s resources are outstretched or exhausted, they enter a defensive mode to preserve the self which is often defensive, aggressive, and may become irrational (Hobfoll et al., 2018). Meanwhile, resource depletion makes it more difficult for individuals to forgive others’ offensive behaviors (Stanton and Finkel, 2012), and when the aversive incidents at work arouse frustration, individuals with resource depletion are unlikely to fully adjust and suppress their aggressive impulses (DeWall et al., 2007; Liu et al., 2015), further causing behavioral problems, such as aggressive behaviors, deviant behaviors, and immoral behaviors (DeWall et al., 2007; Gino et al., 2011; Diestel and Schmidt, 2012).

However, employees who are attacked or harmed by customers will have scruples and fear for organizational punishment; they may not directly carry out reactive aggression toward customers. Instead, they will change the target of aggression and lash out at innocent people around them. Therefore, we propose that it is difficult for employees to suppress their aggressive impulses when they are subjected to customer mistreatment, and because of such impulses they are more likely to vent their anger and aggression toward the innocent people (i.e., coworkers) around them and show more displaced aggressive behaviors. Accordingly, we predict the following hypothesis:

*Hypothesis* 1: Customer mistreatment is positively related to employee displaced aggression.

### The Moderating Role of Interpersonal Sensitivity

In many cases, customer mistreatment is an unconscious and low-intensity behavior with ambiguous intention to harm, such as, urging service progress and bargaining. In view of this kind of customer behavior, how the service personnel define its nature will vary with their own characteristics and cognition. Employees with high interpersonal sensitivity are especially alert and sensitive to the negative attitudes and evaluations of others. They tend to perceive benign social information as rejection or disapproval. High interpersonal sensitivity makes employees to have poor adaptability in interpersonal communication, thus prone to hostile cognition, anger, and aggressive behavior (Lai and Ye, 2015). Therefore, when employees with high interpersonal sensitivity are confronted with some unconscious negative attitudes or behaviors of customers, they are more likely to consider such attitudes or behaviors as an offense to themselves, and thus generating hostility and anger toward customers.

In addition, customer mistreatment is a kind of negative interpersonal interaction that causes service difficulties and psychological pressure to employees. Nevertheless, employees with high interpersonal sensitivity believe that interpersonal difficulties will have particularly serious destructive consequences on their status in the social circle (Marin, 2010). As a result, these employees are more likely to feel inferior and uncomfortable in front of others in this kind of negative interpersonal relationship, and have behaviors such as social avoidance and self-doubt (Davidson et al., 1989; Otani et al., 2008), further leading to social anxiety, depression, and other problems. Furthermore, social anxiety and depression are easy to make individuals demonstrate aggression (Marshall et al., 2011).

Based on the CORs theory, employees with high interpersonal sensitivity are more capable of perceiving customers’ unfriendliness and harm, and then generate negative consequences, such as depression and anger. When suffering customer mistreatment, they deplete relatively more resources for self-control and emotional management, and are more prone to ego depletion effect. Once ego depletion occurs, it is difficult for employees to restrain their aggressive impulses. Because of such impulses, they may get angry at and attack the innocent people around them, showing more displaced aggressive behaviors (Liu et al., 2015). On the contrary, employees with low interpersonal sensitivity are not overly sensitive to other people’s attitudes and behaviors (Boyce and Parker, 1989), as such employees have lower awareness of customer mistreatment and are more likely to ignore the bad feelings brought by customers. Thus, the impact of customer mistreatment on employee displaced aggression may be alleviated in individuals with low interpersonal sensitivity. Taken together, we propose the following hypothesis:

*Hypothesis* 2: Interpersonal sensitivity moderates the relationship between customer mistreatment and employee displaced aggression, such that the positive relationship is stronger when employee interpersonal sensitivity is high rather than low.

### The Moderating Role of Moral Identity

The mistreatment toward employees by customers can be regarded as an “immoral behavior,” which further stimulates the “immoral behavior” of employees toward their colleagues. Therefore, the employees’ own moral characteristics may play a moderating role in this situation. Moral identity is the most powerful intervention factor in the connection between a person’s moral judgment and behavior (Damon and Hart, 1992). When an individual has a high degree of moral identity, it can act as a self-regulating mechanism to stimulate moral behaviors (Erikson, 1964; Blasi, 1984; Hart et al., 1998) and inhibit the individual from producing deviant behaviors (Hardy et al., 2012; Mulder and Aquino, 2013; Skarlicki et al., 2016). It can be seen from this that when mistreated by customers, employees with high moral identity are more inclined to make self-regulation to stimulate their own moral behaviors and inhibit the generation of immoral behaviors.

However, employees’ displaced aggression on colleagues is an aggressive behavior of employees who vent their anger and dissatisfaction on innocent colleagues after they are offended or hurt by customers. Such behavior is contrary to morality and is immoral. It is also an individual deviant behavior. Thus, we predict that individuals with higher moral identity will show lower displaced aggression when experiencing customer mistreatment, while individuals with lower moral identity will show higher displaced aggression when experiencing customer mistreatment. Furthermore, the higher the degree of employees’ moral identity, the more important the status of moral traits in their self-concept, and the higher the sensitivity to moral information for employees, as such employees’ moral identity is easier to be activated by relevant situations (e.g., moral situations), further arouses employees showing more moral behaviors. Meanwhile, the lower the degree of employees’ moral identity, the less important the status of moral traits in their self-concept, and the higher the sensitivity to immoral information for employees, as such employee’ immoral traits have greater potential to be activated by relevant situations (e.g., immoral situations), further leads to employees showing more immoral behaviors (Aquino et al., 2009). Therefore, when facing the same context cues, such as customer mistreatment, employees with different degrees of moral identity may have different behavioral responses.

According to the SCT, when facing customer mistreatment, employees with lower moral identity always ignore organizational service standards, as well as ethical and moral requirements. Consequently, they are stimulated by immoral contexts, and exhibit more immoral behaviors, such as displaced aggression. By contrast, employees with higher moral identity are more sensitive to the surrounding moral information, and have a stronger awareness of cognition and processing of moral information. Accordingly, when suffering customer mistreatment, they are more inclined to ignore the influence of immoral context cues and exhibit behaviors consistent with their own moral standards. Taken together, we predict the following hypothesis:

*Hypothesis* 3: Moral identity moderates the relationship between customer mistreatment and employee displaced aggression, such that the positive relationship is weaker when employee moral identity is high rather than low.

**Figure 1:**
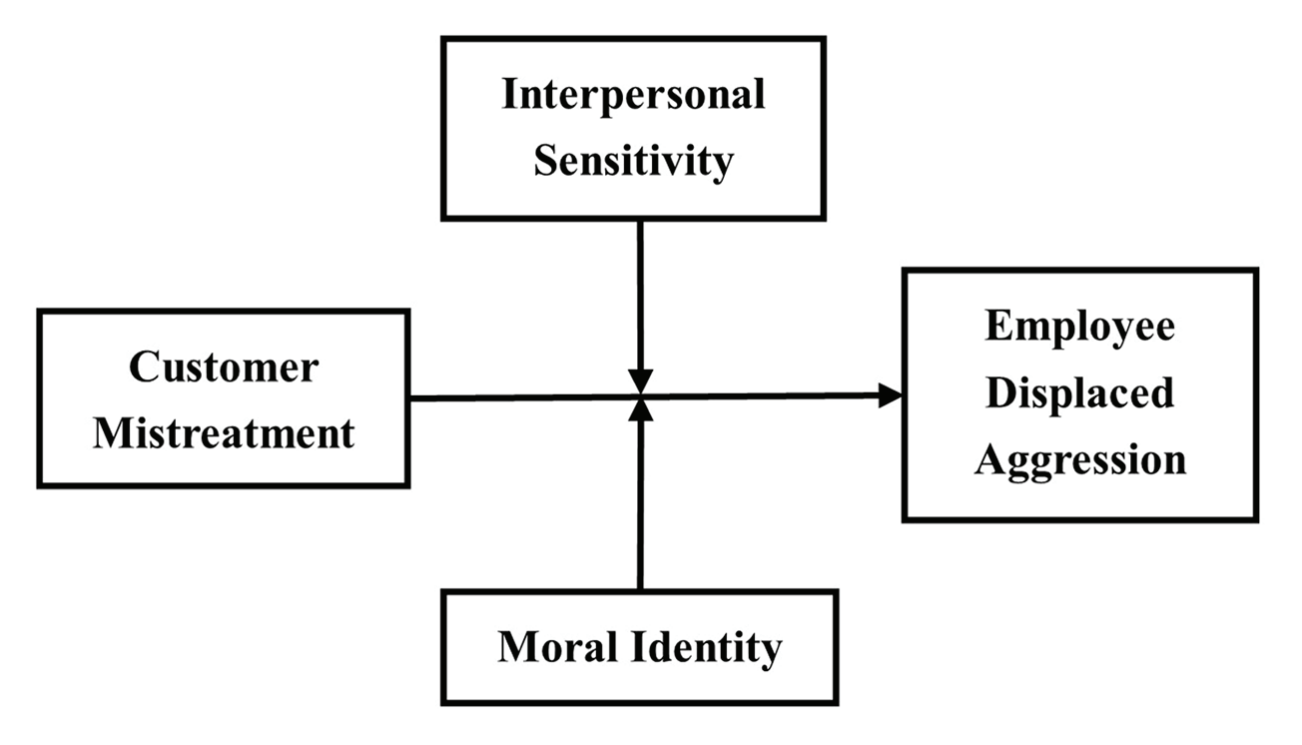
Hypothesized theoretical model.

To sum-up, the theoretical model of this research is shown in Figure 1.

## Materials and Methods

### Participants and Procedure

The samples of this study were taken from the frontline service employees in Chengdu, Zigong, Nanchong, Guangyuan, Mianyang, Deyang, and other cities in China. In order to avoid the influence of common method biases, the research was divided into two stages. In the first stage, we measured demographic-related variables, customer mistreatment, interpersonal sensitivity, and moral identity. A month later, the second stage of measurement was conducted to measure employee displaced aggression.

The questionnaires were implemented using hard copies. The human resource managers distributed the questionnaires to frontline service employees providing face-to-face service to customers, and the employees were assured confidentiality and voluntary participation. Specifically, employees were assured that their managers and organization would not know their individual responses in the survey. The questionnaires were numbered and put into envelops, which were also marked the same value as the questionnaires. In above two measurements, each participant would receive envelopes with an identical number twice to ensure that every participant’s final result from two measurements would not be mixed up with others.

A total of 1,000 questionnaires were distributed in the whole process, and 623 valid questionnaires were recovered, with a recovery rate of 62.3%. The characteristics of the samples are as follows: The majority of respondents were female (56.8%) and unmarried accounting for 72.2% in total; 50.6% of them have bachelor’s degree; the sample age is relatively young, mostly below 25 (65.3%); job tenure mostly less than 1 year, accounting for 45.1%.

### Measures

The scale in this study mainly referred to the relevant scales, which have been used and verified. This study adopted the translation and back-translation procedure to generate the Chinese version of measurements. Our study team translated the original English items into Chinese, and then translated the items back into English, as well as discussed and settled the discrepancies between the original and back-translated versions of the scales to ensure the accuracy of the scale. Meanwhile, according to the suggestions of relevant professionals, the wording was modified to be in line with China’s national conditions so as to ensure the validity and reliability of the measurement tool, after which the Chinese version scales were finalized. The five-point Likert scale was used in the questionnaires, and all the measurements were self-reported.

### Customer Mistreatment

We adopted the scale developed by Chi et al. (2013), which contained three items. The participants were asked to indicate how frequently they had experienced customer mistreatment over the last 2 weeks and indicated their agreement (1 = strongly disagree to 5 = strongly agree). The representative item is “complained about your service performance without reason.” In this study, the Cronbach’ s *α* for the scale was 0.798.

### Displaced Aggression Toward Coworker

Participants were also instructed to complete measures of displaced aggression toward coworker over the last 2 weeks. Participants indicated their agreement (1 = strongly disagree to 5 = strongly agree) with the eight items from Liu et al. (2015). The representative item is “When someone or something made me angry, I took it out on my coworkers.” In this study, the Cronbach’ s *α* for the scale was 0.877.

### Interpersonal Sensitivity

Interpersonal sensitivity was measured by asking employees to indicate how they remember and react to other’s negative feeling and attitude; participants indicated their agreement (1 = strongly disagree to 5 = strongly agree) with the four items developed by Li et al. (2016). The representative item is “It makes me angry if my supervisor or coworkers lie to me.” In this study, the Cronbach’ s *α* for the scale was 0.720.

### Moral Identity

We adopted the scale developed by Aquino and Reed (2002), which contained 10 items. Participants were instructed to complete measures of moral identity and indicated their agreement (1 = strongly disagree to 5 = strongly agree). The representative item is “Being someone who has these characteristics is an important part of who I am.” In this study, the Cronbach’ s *α* for the scale was 0.835.

### Control Variables

We included several control variables in the analyses to rule out the possible confounding effects. First, we controlled for employees’ gender (1 = male; 2 = female) and age (1 = below 25 years; 2 = 26–30 years; 3 = 31–35 years; 4 = 36–40 years; 5 = above 41 years), since women are more likely to suffer mistreatment in the work situation (Lim and Lee, 2011), as well as age and gender have been shown to relate positively with moral identity (Reed and Aquino, 2003; Skarlicki et al., 2008). Second, we included marital status (1 = unmarried; 2 = married) as control variables due to marriage theory (Becker, 1981); employees who get married become more responsible, mature, and inclusive, thus it’s more likely to show greater tolerance and understanding when suffering customer mistreatment. Third, we included employee tenure (1 = below 1 year; 2 = 1–3 years; 3 = 4–6 years; 4 = 7–10 years; 5 = above 11 years) and education level (1 = high school and below; 2 = college diploma; 3 = bachelor degree; 4 = master degree and above) as control variables because job tenure affects the learning opportunity and practice skills of staff to deal with customer problems and demands (Schmidt et al., 1986; Wang et al., 2011), and education level may affect employees’ experience and knowledge of how to deal with different types of customers (Wen and Hou, 2015). Finally, in Chinese characteristic ownership structure, different industries are variant in service concepts, service standards, service norms, service procedures, and even the service environment, as such, employees’ attachment and involvement to the organization is also different (Zhang and Liao, 2017). All these control variables were self-reported.

## Results

### Assessment of Common Method Bias

Harman’s one-factor test can be used to address the common method bias problem (Podsakoff and Organ, 1986), which was to do factor analysis for all items in the questionnaire. Accordingly, we found that the first factor explained only 23.297% of the variance without rotation, and that both the dependent and independent variables were loaded on different factors. Because a single factor neither does not appear nor does it appear that a single factor explains the majority variance, in this paper, common method bias does not appear to have a substantial impact on the present study.

### Confirmatory Factor Analysis

Customer mistreatment, interpersonal sensitivity, moral identity, and displaced aggression are the four main variables in the theoretical model of this study. In order to test the discriminant validity among the variables, we used the Lisrel 8.70 statistical software to carry out confirmatory factor analysis. The results are shown in Table 1. Among them, the fit index of the four-factor model is at an acceptable level (*χ* ^2^ = 1,135.59, *df* = 269, NNFI = 0.92, CFI = 0.93, RMR = 0.05, RMSEA = 0.07) and superior to the three-factor, two-factor, and single-factor models.

**Table 1:** Results of confirmatory factor analysis.

	*χ* ^2^	*df*	RMSEA	CFI	NNFI	RMR
Model 1 (four factors: CM, IS, MI, and DA)	1135.59	269	0.07	0.93	0.92	0.05
Model 2 (three factors: CM + IS, MI, and DA)	1794.29	272	0.10	0.89	0.88	0.08
Model 3 (three factors: CM + MI, IS, and DA)	1783.71	272	0.10	0.88	0.86	0.08
Model 4 (three factors: CM, IS + MI, and DA)	1833.67	272	0.10	0.89	0.87	0.08
Model 5 (two factors: CM + IS and MI + DA)	4239.08	274	0.15	0.77	0.75	0.13
Model 6 (two factors: CM + MI and IS + DA)	2349.91	274	0.11	0.84	0.82	0.10
Model 7 (two factors: CM + IS + MI and DA)	2507.48	274	0.11	0.83	0.81	0.11
Model 8 (two factors: CM + DA and IS + MI)	2362.98	274	0.11	0.84	0.82	0.10
Model 9 (one factor: CM + IS + MI + DA)	4669.03	275	0.16	0.73	0.70	0.13

*N* = 623; CM = customer mistreatment; IS = interpersonal sensitivity; MI = moral identity; DA = displaced aggression; and + represents the combination of two factors into one variable.

### Descriptive Statistics

The mean value, standard deviation, and correlation coefficient of the variables are shown in Table 2. Among them, customer mistreatment and interpersonal sensitivity are both significantly positively correlated to displaced aggression, while moral identity and displaced aggression are significantly negatively correlated, which are consistent with the research expectations. However, regarding the control variables, only education level positively correlated to interpersonal sensitivity and moral identity, while gender, age, marital status, tenure, and industry are not statistically correlated.

**Table 2:** Descriptive statistics and correlation analysis.

	Mean	SD	Skewness	Kurtosis	1	2	3	4	5	6	7	8	9
1.Gender	1.570	0.496	−0.276	−1.930									
2.Age	1.730	1.232	1.649	1.475	0.089^*^								
3.Marital status	1.280	0.448	0.995	−1.013	0.121^**^	0.752^***^							
4.Education level	2.360	0.945	−0.334	−1.166	−0.139^**^	−0.492^***^	−0.447^***^						
5.Tenure	1.790	0.929	1.440	2.345	0.083^*^	0.649^***^	0.564^***^	−0.333^***^					
6.Industry	3.100	1.306	−0.337	−1.242	0.023	0.188^***^	0.173^***^	−0.251^***^	0.145^***^				
7.Customer mistreatment	2.460	0.853	0.369	−0.010	−0.049	−0.050	−0.014	0.015	0.008	−0.030			
8.Interpersonal sensitivity	3.521	0.712	−0.708	0.917	−0.052	−0.034	−0.042	0.091^*^	−0.034	−0.007	0.094^*^		
9.Moral identity	3.932	0.536	−0.482	0.372	0.056	−0.023	−0.059	0.209^***^	0.009	−0.059	−0.127^**^	0.025	
10.Displaced aggression	1.816	0.657	0.823	0.510	−0.019	0.040	0.011	−0.044	−0.006	0.044	0.288^***^	0.257^***^	−0.289^***^

*N* = 623.

* *p* < 0.05;

** *p* < 0.01;

*** *p* < 0.001.

### Hypotheses Testing

In order to test the relationship between customer mistreatment and employee displaced aggression, as well as the moderating effect of employees’ interpersonal sensitivity and moral identity on this relationship, we used SPSS 23.0 to conduct the hierarchical multiple regression test. The test results are shown in Table 3. According to the analysis results of Model 2, customer mistreatment is positively related to displaced aggression, and Hypothesis 1 is supported.

**Table 3:** Results of regression analysis.

Variables	Displaced aggression						
	Model 1	Model 2	Model 3	Model 4	Model 5	Model 6	Model 7
Control variables
Gender	−0.023	−0.008	0.000	−0.001	0.013^*^	0.013	0.021
Age	0.082	0.121	0.110	0.093	0.147	0.141	0.119
Marital status	−0.040	−0.054	−0.050	−0.043	−0.07	−0.074	−0.064
Education level	−0.032	−0.027	−0.049	−0.046	0.042	0.049	0.027
Tenure	−0.051	−0.071	−0.065	−0.056	−0.055	−0.045	−0.035
Industry	0.035	0.043	0.039	0.037	0.038	0.034	0.030
Independent variable
Customer mistreatment		0.295^***^	0.273^***^	0.274^***^	0.263^**^	0.263^***^	0.241^***^
Moderators
Interpersonal sensitivity			0.235^***^	0.247^***^			0.239^***^
Moral identity					−0.263^***^	−0.276^***^	−0.273^***^
Interaction
CM × IS				0.082^*^			0.064
CM × MI						−0.148^***^	−0.131^***^
CM × IS × MI							−0.029
*F*	0.675	8.990^***^	13.22^***^	12.337^***^	14.245^***^	14.777^***^	15.462^***^
*R* ^2^	0.007	0.093^***^	0.147^***^	0.153^*^	0.157^***^	0.178^***^	0.233^**^
▵*R* ^2^	0.007	0.086^***^	0.054^***^	0.006^*^	0.064^***^	0.022^***^	0.020^**^
▵*F*	0.675	58.501^***^	38.948^***^	4.642^*^	46.388^***^	16.209^***^	5.302^**^

*N* = 623, CM = customer mistreatment; IS = interpersonal sensitivity; and MI = moral identity.

* *p* < 0.05;

** *p* < 0.01;

*** *p* < 0.001.

**Figure 2:**
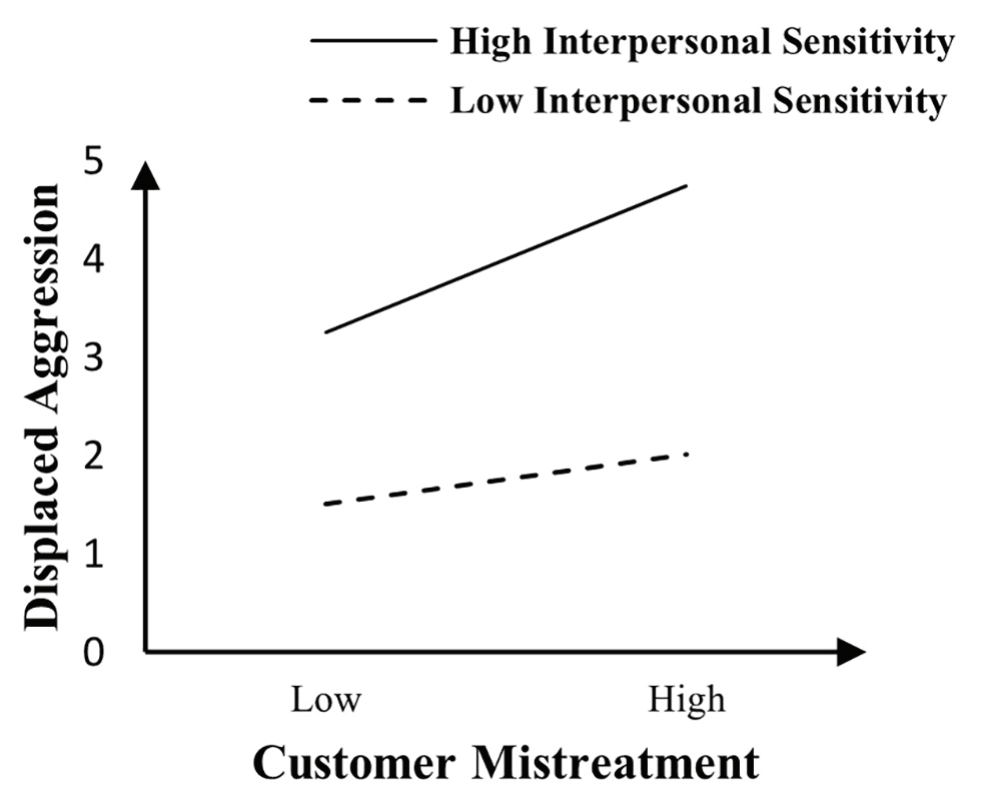
Interpersonal sensitivity as a moderator of the relationship between customer mistreatment and displaced aggression toward coworkers.

**Figure 3:**
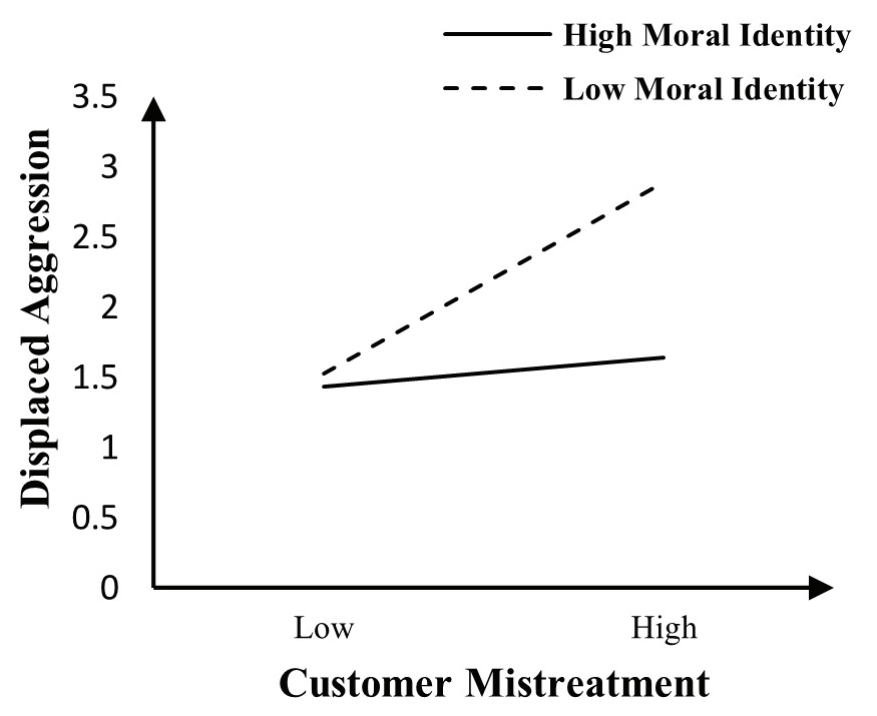
Moral identity as a moderator of the relationship between customer mistreatment and displaced aggression toward coworkers.

In order to test the moderating effect, we centralized customer mistreatment, interpersonal sensitivity, and moral identity, and performed regression analysis after multiplication. The analysis results of Model 4 show that interpersonal sensitivity exacerbating the influence of customer mistreatment on displaced aggressive behaviors (*β* = 0.082, *p* < 0.05). Hypothesis 2 is supported. The specific moderating effect diagram is shown in Figure 2. The analysis results of Model 6 show that moral identity buffering the influence of customer mistreatment on displaced aggression toward coworkers (*β* = −0.148, *p* < 0.001). Hypothesis 3 is supported. The specific moderating effect diagram is shown in Figure 3.

Both Figures 2, 3 show that when employees experience increased customer mistreatment, their displaced aggressive behaviors toward their coworkers will enhance. Figure 2 shows that the higher the interpersonal sensitivity of employees, the more likely they are to generate displaced aggressive behaviors when they are subjected to customer mistreatment. Conversely, when their interpersonal sensitivity is lower, the relationship between customer mistreatment and displaced aggressive behaviors will be weakened. Figure 3 shows that the higher the moral identity of employees, the lower the possibility that employee displaced aggressive behaviors will occur when they are subjected to customer mistreatment. Conversely, when the moral identity is lower the positive relationship between customer mistreatment and displaced aggression will be enhanced. Model 7 shows that a three-way interaction effect is not significant (*β* = −0.029, *p* > 0.05), that is, in the explanation of customer mistreatment, interpersonal sensitivity, and moral identity to displaced aggressive behaviors, the interactive moderating effect among the three factors does not exist.

## Discussion

### Research Conclusions

Based on the CORs theory and SCT, this study explores the relationship between customer mistreatment and employee displaced aggression toward colleagues, as well as the moderating effect of interpersonal sensitivity and moral identity on the above relationship. The results show that customer mistreatment has a positive impact on employee displaced aggression. Interpersonal sensitivity plays a positive moderating role between customer mistreatment and employee displaced aggression, such that the higher the interpersonal sensitivity of employees, the more intense their displaced aggression toward coworkers when they are subjected to customer mistreatment. Moral identity plays a negative moderating role between customer mistreatment and employee displaced aggression, such that employees with high moral identity generate less displaced aggression when facing customer mistreatment.

### Theoretical Implications

The current study offers several theoretical contributions. First, the influence mechanism of customer mistreatment on employee displaced aggression is studied. Existing studies on the adverse consequences of customer mistreatment mostly focus on the aspects of employee-customer and employee-organization (Boswell and Olson-Buchanan, 2004; Wang et al., 2011; Baranik et al., 2017), and the research on the influence of employee-coworkers is very deficient. Through model construction, this paper connects the customer mistreatment with employee displaced aggression toward colleagues, further effectively reveals the internal mechanism between the two, as well as aims to be a supplement for previous study.

Besides, this study has introduced the moderating effect of interpersonal sensitivity based on CORs theory, and enriched the research on the intervention mechanism of customer mistreatment and its consequences. It has revealed that the interpersonal sensitivity could be regarded as a kind of individual resource, which has exacerbating effect on the relationship between customer mistreatment and employee displaced aggression; meanwhile, confirmed the previous researchers’ view that “high interpersonal sensitivity generates more intense explicit aggression” (Lai and Ye, 2015, p. 112).

Further, in light of SCT, this study has revealed moral identity as another key boundary condition of the influence mechanism of customer mistreatment on employee displaced aggression. The results showed that the higher the degree of employees’ moral identity, the weaker the positive influence of customer mistreatment on employee displaced aggression, as such, moral identity buffering the effect of customer mistreatment on employee displaced aggression. It not only provides a new idea for the research of the intervention of individual characteristics in customer mistreatment and its behavioral consequences in the field of organizational behavior, but it also confirms that the previous research conclusions that “employees with high moral identity, even under the influence of corporate hypocrisy, will not easily find excuses to engage in unethical behavior” (Zhao and Zhou, 2017, p. 18).

Unfortunately, the three-way interaction (customer mistreatment × interpersonal sensitivity × moral identity) was not statistically significant, possibly due to limitations in data collection. Although we collected data from different industries in different cities, in order to eliminate the data limitations, these cities are all belong to Sichuan Province of China, and the regional limitations may be impair data validity. In future studies, we will pay attention to this issue and widen data collecting scale (e.g., collecting some samples in other provinces instead of being limited to one province).

In addition, previous research on customer mistreatment has focused on call centers (Skarlicki et al., 2008; Wang et al., 2013), which are not consistent with the customer-employee face-to-face service situation in most service organizations. This study takes frontline service employees as the research sample from finance, service, medicine, education, and other industries, and aims at the customer-employee face-to-face service form, so as to enlarge the research scope of customer mistreatment. Meanwhile, the research results provide more comprehensive and extensive guidance for the customer-employee service industry.

It’s worth noting that statistic results of control variables, we found non-significant associations between control variables and our constructs of interest in correlations and regressions, possible reason is the form of service. As mentioned earlier, Skarlicki et al. (2008) and Wang et al. (2011) recruited participants both from call centers. Nevertheless, our data come from face-to-face service staff, which is significantly different from telephone service in terms of service forms and service situation. Our research model is more likely influenced by some specific mistreatment from customers, as well as employees’ education level and individual traits (i.e., interpersonal sensitivity and moral identity), rather than employees’ gender, age, marital status, tenure, and industries.

### Practical Implications

This study provides several practical implications. First, we explained the impact of “customer behavior” on “employee behavior,” so that company managers can understand that customer mistreatment leads to employee displaced aggression toward colleagues. Hence, enterprises can create a civilized and harmonious service atmosphere to reduce the frequency of customer mistreatment; on the other hand, by fostering an atmosphere of teamwork and mutual assistance, employees can obtain more team support to reduce resource consumption and aggressive behaviors toward colleagues.

Second, in view of the intervention effect of employees’ interpersonal sensitivity, it is suggested that managers should pay more attention to evaluating candidates’ interpersonal sensitivity in the recruitment process and choose employees with lower interpersonal sensitivity for frontline service. Meanwhile, they should pay attention to the psychological state of employees and alleviate their psychological stress and social anxiety through expert consultation and training.

Third, the management should institute a formal written zero-tolerance policy for customer mistreatment, distinguishing reasonable from unreasonable customers’ demands (Choi et al., 2014). Furthermore, managers should ensure that they are providing quality customer services, and they are soliciting frequent feedbacks from clients to detect service failures (Sommovigo et al., 2019a).

Finally, enterprises should attach importance to provide their employees with regularly moral education and psychological resilience training. Moral education could strengthen their moral concepts, consolidate their inner moral standards, promote the consistency of their own behaviors, and reduce the occurrence of immoral behaviors. Meanwhile, psychological resilience training programs (Meiklejohn et al., 2012) aimed at improving employees’ skills to face customer interactions, respond to customers’ requests, and foster service providers’ emotion regulation skills, coping strategies, and relational management skills (Sommovigo et al., 2019a).

### Limitations and Future Research Directions

In general, this study makes some theoretical and practical contributions, but there are still some limitations to be improved. First, this study only explores the relationship between customer mistreatment and employee displaced aggression from the perspective of resources, future studies can explore the above relationship from different theoretical perspectives.

Second, our research focuses on the intervention research of customer mistreatment and its adverse consequences from the perspective of individual differences. Subsequent researches could study the adjustment mechanism from the perspectives of situational and individual‐ situational interaction.

Third, the cross-sectional data used in this study may not be sufficient to judge the causal relationship between variables (Zhang and Long, 2017). Future research should use the daily diary method to collect panel data to better test and judge the causal relationship between variables. Meanwhile, the sample of our study is from Sichuan province, as such, the representativeness of sample is limited (Wen and Hou, 2015). In the future research, the sample selection range ought to be expanded to improve the universality of the research.

Forth, Sliter et al. (2012) analyzed the unique and combined effects of two sources of incivility (customer and coworker) on objective sales performance and withdrawal behaviors (absenteeism and tardiness), confirming the importance of considering combined effects. We should acknowledge that future research should analyze the differences of receiving mistreatment from one single source versus multiple sources.

Finally, Shao and Skarlicki (2014) conducted a field study of customer service employees (*N* = 213) working in the same hotel chain in China and Canada, found that customer mistreatment was predicted to be associated with different reactions among North American and East Asian employees. As such, cultural contexts may affect employees’ reactions to customer mistreatment. Whereas, due to limited resources, the samples of this study were all from China as well as focused on displaced aggression within the Chinese context, future study should consider possible differences related to cultural aspects because employees from different countries may react differently to customer mistreatment (e.g., Shao and Skarlicki, 2014; Sommovigo et al., 2020).

## Data Availability Statement

The raw data supporting the conclusions of this article will be made available by the authors, without undue reservation.

## Ethics Statement

The studies involving human participants were reviewed and approved by the Institutional Review Board, School of Business Administration, Southwestern University of Finance and Economics. The patients/participants provided their written informed consent to participate in this study.

## Author Contributions

FL was responsible for the designing and writing. GC was responsible for the data analysis. YL was responsible for English language editing. All authors contributed to the article and approved the submitted version.

### Conflict of Interest

The authors declare that the research was conducted in the absence of any commercial or financial relationships that could be construed as a potential conflict of interest.
